# Long non-coding RNA HIF1A-AS2 modulates the proliferation, migration, and phenotypic switch of aortic smooth muscle cells in aortic dissection via sponging microRNA-33b

**DOI:** 10.1080/21655979.2022.2041868

**Published:** 2022-02-25

**Authors:** Kai Zhang, Yujuan Qi, Meng Wang, Qingliang Chen

**Affiliations:** a Department of Cardiac Surgery, Tianjin Chest Hospital, Tianjin, China; b Department of Cardiac ICU, Tianjin Chest HospitalTianjin, China , Tianjin China; c Department of Cardiac ICU, Tianjin Chest Hospital, Tianjin, China

**Keywords:** Aortic dissection, HIF1A-AS2, SMCs, migration, phenotypic switching

## Abstract

Aortic dissection (AD), also known as aortic dissecting aneurysm, is one of the most common and dangerous cardiovascular diseases with high morbidity and mortality. This study was aimed to investigate the functional role of long non-coding RNA Hypoxia-inducible factor 1 alpha-antisense RNA 2 (lncRNA HIF1A-AS2) in AD. An *in vitro* model of AD was established by platelet-derived growth factor-BB (PDGF-BB)-mediated human aortic Smooth Muscle Cells (SMCs). HIF1A-AS2 expression in human AD tissues was determined by quantitative real-time PCR (qRT-PCR) and fluorescence in situ hybridization (FISH) assays, followed by investigation of biological roles of HIF1A-AS2 in AD development by Cell Counting Kit-8 (CCK-8), immunofluorescence, and transwell assays. Additionally, the correlation between HIF1A-AS2, miR-33b, and high mobility group AT-hook2 (HMGA2) were identified by RNA immunoprecipitation (RIP), RNA pull-down and luciferase reporter assays. Results showed that HIF1A-AS2 was obviously increased, while the contractile-phenotype markers of vascular SMCs were significantly decreased in human AD tissues, when compared to normal tissues. Inhibition of HIF1A-AS2 attenuated SMCs proliferation and migration, whereas enhanced the phenotypic switch under the stimulation of PDGF-BB. Results from RIP, RNA pull-down and luciferase reporter assays demonstrated that miR-33b directly bound with HIF1A-AS2, and HIF1A-AS2 silencing suppressed the expression of HMGA2, which was induced by miR-33b inhibitor. In conclusion, knockdown of HIF1A-AS2 suppressed the proliferation and migration, while promoted the phenotypic switching of SMCs through miR-33b/HMGA2 axis, which laid a theoretical foundation for understanding the development of AD and shed light on a potential target for AD treatment.

## Graphical abstract

**Figure:**
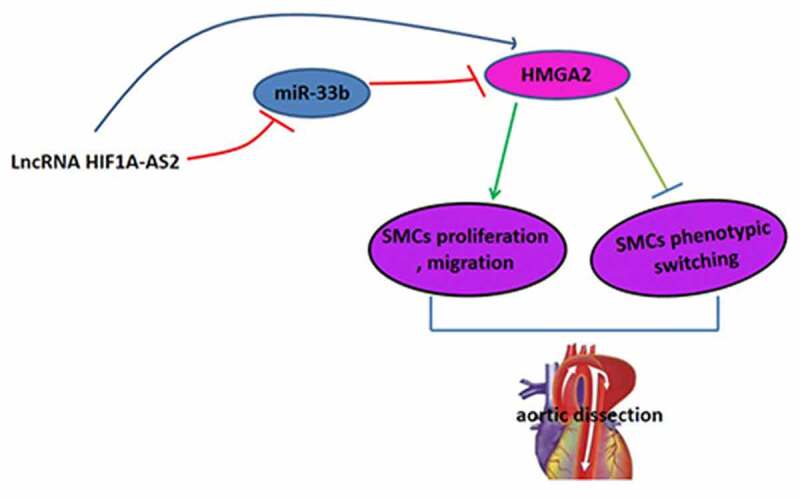

## Introduction

Aortic dissection (AD) is a fetal cardiovascular diseases with high mortality even when treatment with surgery, drug, and endovascular repair [^1–3^]. Thus, it is necessary to find effective therapeutic targets at the molecular level to prevent the pathological progression of AD. Despite significant progress has been made, the underlying mechanism is still not fully understood, which limits the identification of potential targets. The molecular pathogenesis of AD is related to the structural and functional changes of smooth muscle cells (SMCs) in the ascending aorta, including SMC proliferation, migration, and phenotypic switching, which induces the formation of the AD [4,5]. Previous studies have demonstrated that vascular SMCs phenotypic switching causes the pathophysiological changes of many cardiovascular diseases, including atherosclerosis, hypertension, and AD [6,7]. As previous studies shown, α-smooth muscle actin (α-SMA), smooth muscle protein-22α (SM22α), calponin, and myosin heavy chain 11 (MYH11) are specific markers of contractile vascular SMCs. Although there are many hypotheses about the pathogenesis of AD, the exact cause is still unclear [8].

Long non-coding RNAs (LncRNAs), longer than 200 nt, are also the linear RNAs that distributed in the nucleus or cytoplasm [9]. A large number of sequencing results have found that lncRNAs are differentially expressed in cardiovascular diseases tissues and normal tissues, which lays the foundation for lncRNAs to become the serum biomarkers [^10–12^]. The function of lncRNA, which has been studied more frequently, is mainly through competitive binding with miRNA, so as to relieve the inhibitory effect of miRNA on target genes [13]. The mechanism of this competitive endogenous RNA has been discovered in the process of many diseases and has become a current research hotspot [14,15]. However, the current research on lncRNAs in AD is relatively rare and superficial.

As an antisense transcript of HIF-1α, Hypoxia-inducible factor 1 alpha-antisense RNA 2 (HIF1A-AS2) has been implicated in the hypoxia-associated tumor processes [16,17]. *In vitro* studies have revealed the role of HIF1A-AS2 in cell proliferation, migration, and apoptosis of various tumors, including glioblastoma [18], renal carcinoma [19], and lung cancer [20]. More importantly, Li P. et al. reported that HIF1A-AS2 is involved in atherosclerosis progression through promotion of SMCs proliferation and inhibition of SMCs apoptosis [16]. However, the mechanism underlying the effect of HIF1A-AS2 in SMCs proliferation, migration, and phenotypic switching in AD is not well understood.

In the current study, we hypothesized that HIF1A-AS2 modulates PDGF-BB-induced SMCs proliferation, migration, and phenotypic switching. The purpose of this study was to investigate the expression of HIF1A-AS2 in human AD samples, detect the expressions of miR-33b and HMGA2 and the alterations in SMCs proliferation, migration, and phenotypic switching after HIF1A-AS2 knockdown, and explore the underlying molecular mechanisms.

## Materials and methods

### Human aortic samples

Eight human AD tissues and adjacent aortic tissues were obtained from the patients who underwent surgery in Tianjin Chest Hospital. The adjacent aortic tissues were used as the control group [21]. The AD diagnosis was confirmed by computed tomography angiography. A portion of the sample was stored at −80°C for further use. This study was approved by the Ethics Committees of Tianjin Chest Hospital (Approval No. TCH20180104096), and both patients were aware of the study content and signed informed consent.

### Cell culture and cell model construction

Primary SMCs were collected from thoracic aorta media of patients who received from aortic valve replacements [22]. The cells were cultured in smooth muscle cell medium containing with 10% fetal bovine serum (FBS; Gibco, Carlsbad, USA) and then maintained at 37°C with 5% CO2. SMCs induced by PDGF-BB were used to simulate AD cell model *in vitro* [23,24]. When SMCs were cultured to the logarithmic growth stage, 0.25% trypsin was added to digest the cells and starved for 24 h. Then, the cells were processed by PDGF-BB (20 ng/ml) for 12 h for subsequent studies.

### Cell transfection

Small interfering RNA for HIF1A-AS2 (si-HIF1A-AS2) and the scramble siRNA used as the negative control (si-NC) were synthesized by GenePharma (Shanghai, China). The miR-33b mimic, inhibitor, and the negative controls were purchased from GenePharma (Shanghai, China). SMCs at 2 × 10^5^ cells/well were co-transfected with miR-33b mimic, inhibitor, or si-HIF1A-AS2 using Lipofectamine 2000 reagent (Invitrogen, USA) [25].

### Fluorescence in situ hybridization (FISH)

The fuorescence-conjugated HIF1A-AS2 probes were generated by GenePharma Company (Shanghai, China). After immobilization and permeabilization, the paraffin-embedded aorta tissue slices were hybridized with Cy3-labeled HIF1A-AS2 probes. Subsequently, the sections were incubated with DAPI to stain cell nuclei. An Olympus fluorescence microscope was applied to capture the images [23].

### RNA isolation and quantitative reverse transcription-PCR (qRT-PCR)

Total RNAs were isolated from the SMCs and human AD tissues by means of the TRIzol reagent (Invitrogen). cDNA was synthesized by the PrimeScript RT Reagent Kit (TaKaRa, Dalian, China). SYBR Premix Ex Taq (TaKaRa) was carried out to analyze the qRT-PCR. U6 and GAPDH were used as a quantitative control [26]. The relative expression was determined by the 2− ΔΔCt method.

### Western blotting assay

The proteins were extracted from tissues and SMCs. The protein concentration was determined with the BCA kit. Equal amounts of protein were electrophoretically separated by sodium dodecyl sulfate/polyacrylamide gel electrophoresis (SDS/PAGE) in 10% gels and then transferred onto the polyvinylidene difluoride (PVDF) membranes. Afterward, the membranes were blocked by incubation with 5% skimmed milk, prior to incubation of the membranes with primary antibodies against HMGA2, α-SMA, SM22α, calponin, MYH11, and GAPDH, followed by the secondary antibodies. The immune complexes were visualized by western luminescent detection kit and the density was quantified using the Quantity One software (v1.8.0) [27].

### RNA immunoprecipitation (RIP) assay

As previously described [28], SMCs were harvested in lysis buffer, and the lysate was immunoprecipitated with anti-Ago2 or anti-IgG. Afterward, the precipitation of RNA was extracted. QRT-PCR was used to determine the extracted RNA precipitates.

### Luciferase reporter assay

The 3'UTR of HIF1A-AS2 containing the binding sites of miR-33b were inserted into luciferase vector pMirGLO, named pMirGLO-HIF1A-AS2-Wild-Type (HIF1A-AS2-WT), pMirGLO-HIF1A-AS2-Mutated (HIF1A-AS2-MuT). The pMirGLO-HMGA2-wild-type (HMGA2-WT), pMirGLO-HMGA2-Mutated (HMGA2-MuT) were constructed as described above. The reporter vectors and miR-33b mimic were con-transfected into SMCs. After transfection for 48 h, the luciferase activities were measured using the Dual-Luciferase Assay Manual (Promega) [29].

### RNA pull-down assay

The commercially synthesized biotin-labeled HIF1A-AS2 was purchased from Genepharma (Shanghai, China) and transfected into SMCs for 48 h. Then, the cell lysate was incubated with Dynabeads M-280 Streptavidin (Sigma, MO, USA) according to the manufacturer’s instructions [30]. TRIzol was used to elute and purify the interacted RNA complex, and qRT-PCR was used to measure the expression level of miR-33b.

### Cell Counting Kit-8 (CCK-8) assay

1 × 10^5^ cells/well SMCs were seeded in 96-well plates and cultured for 12, 24, 36, and 48 h. 10 μl of CCK-8 solution was added to each well and incubated for another 4 h at 37°C. Absorbance was determined at 450 nm using Multiskan FC Microplate Photometer (Thermo Fisher Scientific, USA) [22].

### Immunofluorescence assay

SMCs were fixed with 4% polyoxymethylene, permeabilized with 0.2% Triton X-100, and then blocked by incubation with 5% donkey serum. The primary antibodies for Ki-67 was applied, followed by the appropriate Cy3-conjugated secondary antibody. The relative percentage of proliferating SMCs was defined as follows: positive cells/total cells (DAPI-stained cells) [31]. The numbers of positively stained cells were counted from five randomly non-overlapping fields of five independent experiments.

### Cell migration assay

1 × 10^5^ SMCs were plated into the upper chamber of a transwell insert, and medium supplemented with 10% FBS was used as an attractant in the lower chamber. After incubation for 48 h, the migrated cells were fixed with methanol, stained with crystal violet, imaged, and counted [32].

### Statistical analysis

Data were expressed as mean ± standard deviation (SD) at three times. Statistical analyses were determined using Tukey’s post hoc tests via analysis of variance or Student’s t-test, with comparisons among multiple groups or two groups. All statistical analyses were done using SPSS 17.0 statistical software and GraphPad Prism 5. P < 0.05 was regarded as statistically significant.

## Results

This present study was aimed to explore the functional role of HIF1A-AS2 in AD development. An *in vitro* model of AD was established by PDGF-BB-mediated human aortic SMCs, and results showed that HIF1A-AS2 was obviously increased in human AD tissues and knockdown of HIF1A-AS2 suppressed the proliferation and migration, while promoted the phenotypic switching of SMCs through miR-33b/HMGA2 axis, which laid a theoretical foundation for understanding the development of AD and might provide novel strategies for AD therapy.

### HIF1A-AS2 was highly expressed and contractile-phenotype markers were lowly expressed in human aortic tissues

**Figure 1.**
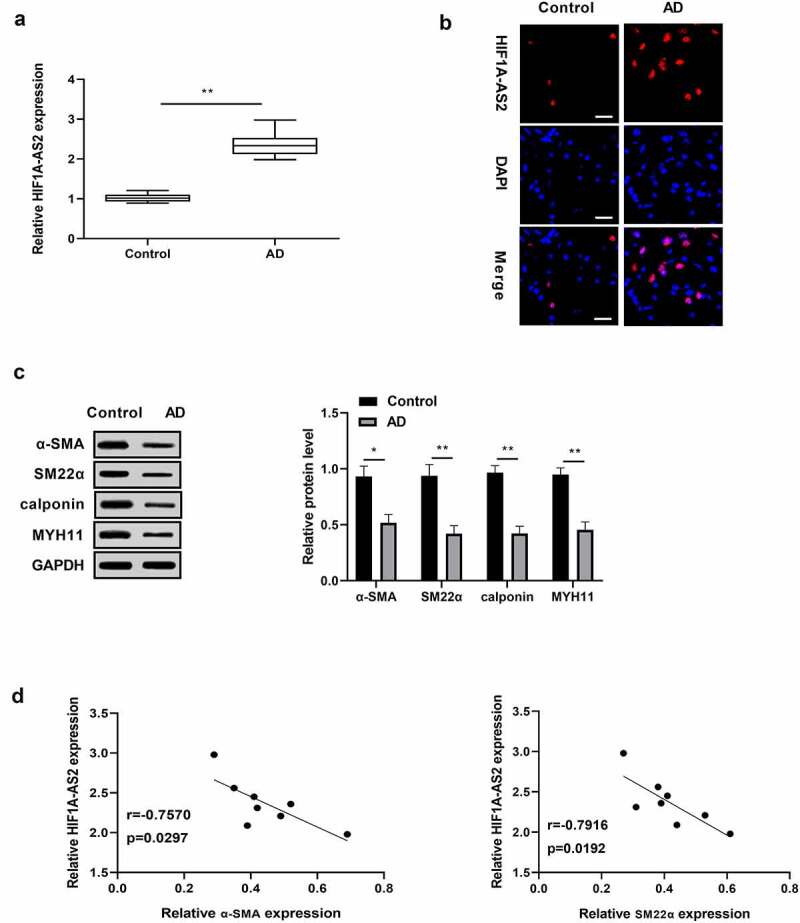
Expression levels of HIF1A-AS2 and contractile phenotypes markers in human aortic tissues. (a) HIF1A-AS2 was elevated in AD tissues (n = 8) versus to that of control (n = 8) by qRT-PCR and (b) FISH assay (Scale bar = 25 μm). (c) The SMC contractile phenotypes markers (α-SMA, SM22α, calponin, and MYH11) were reduced in human AD tissues relative to that of control by Western blotting assay. (d) The correlation between HIF1A-AS2 and contractile phenotype markers (α-SMA, SM22α) were negatively. *P < 0.05, **P < 0.01.

To investigate whether HIF1A-AS2 is aberrantly expressed in AD, HIF1A-AS2 expression in human AD tissues were determined by qRT-PCR and FISH assays, and results discovered that HIF1A-AS2 was dramatically increased in the AD tissues, relative to normal tissues (Figure 1(a,b)). Western blotting analysis showed that the markers of the SMC contractile phenotypes were under expressed in human AD tissues in comparison with the normal tissues (Figure 1(c)). Moreover, there was a negative correlation between HIF1A-AS2 and contractile-phenotype markers (Figure 1(d)). The findings indicated that HIF1A-AS2 might play a key regulatory role in the phenotypic switching of AD.

### Knockdown of HIF1A-AS2 suppressed SMCs proliferation and migration, and promoted phenotypic switching

**Figure 2.**
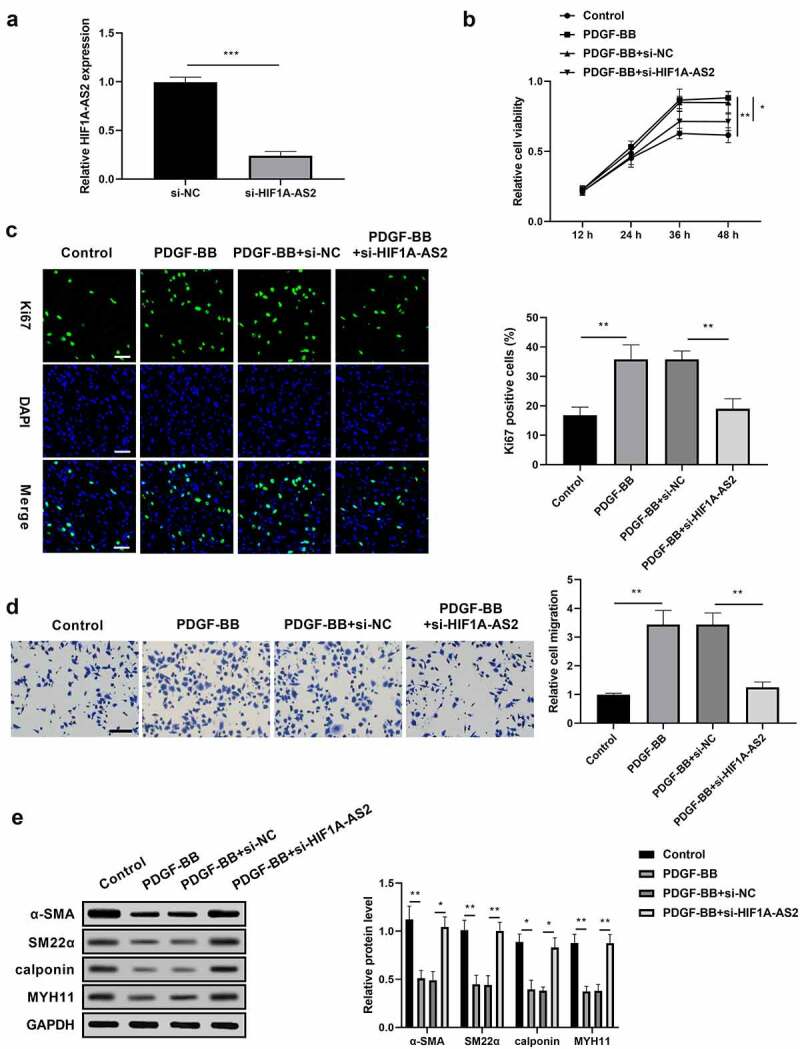
Knockdown of HIF1A-AS2 suppressed SMCs proliferation and migration, while promoted phenotypic switching. (a) The expression of HIF1A-AS2 was detected by qRT-PCR in si-HIF1A-AS2-transfection SMCs. (b) The effect of si-HIF1A-AS2 on SMCs viability was determined by CCK-8 assay. (c) The effect of si-HIF1A-AS2 on Ki67 positive cells was measured by Immunofluorescence assay (Scale bar = 50 μm). (d) The effect of si-HIF1A-AS2 on cell migration was detected by transwell assay (Scale bar = 50 μm). (e) The effect of si-HIF1A-AS2 on α-SMA, SM22α, calponin, and MYH11 protein levels were examined by Western blotting. *P < 0.05,** *P* < 0.01, *** *P*< 0.001.

To determine the functional role of HIF1A-AS2 in AD development, the effects of HIF1A-AS2 on SMCs during vascular pathophysiological responses of the aortic wall were evaluated. SMCs were treated with si-HIF1A-AS2 prior to PDGF-BB treatment. As shown in Figure 2(a), the successfully transfection efficiency of HIF1A-AS2 was verified by qRT-PCR. Results from CCK-8 assay displayed that knockdown of HIF1A-AS2 inhibited the viability of SMCs induced by PDGF-BB stimulation (Figure 2(b)). Moreover, si-HIF1A-AS2 significantly reduced the immunofluorescence of Ki-67, a nuclear protein, acting as a marker for cell proliferation, under the conditions of PDGF-BB stimulation (Figure 2(c)). These findings suggested that down-regulation of HIF1A-AS2 attenuated SMCs proliferation induced by PDGF-BB stimulation. Transwell migration assay discovered that the migration of SMCs upon PDGF-BB treatment was obviously increased, while decreased as HIF1A-AS2 was down-regulated (Figure 2(d)), which indicated that silence of HIF1A-AS2 suppressed PDGF-BB-induced cell migration. In addition, HIF1A-AS2 silencing promoted the expressions of α-SMA, SM22α, calponin, and MYH11 in SMCs, which was reduced by PDGF-BB stimulation (Figure 2(e)). These results suggested that HIF1A-AS2 knockdown suppressed SMCs proliferation and migration, and promoted phenotypic switching.

### HIF1A-AS2 directly interacted with miR-33b in SMCs

**Figure 3.**
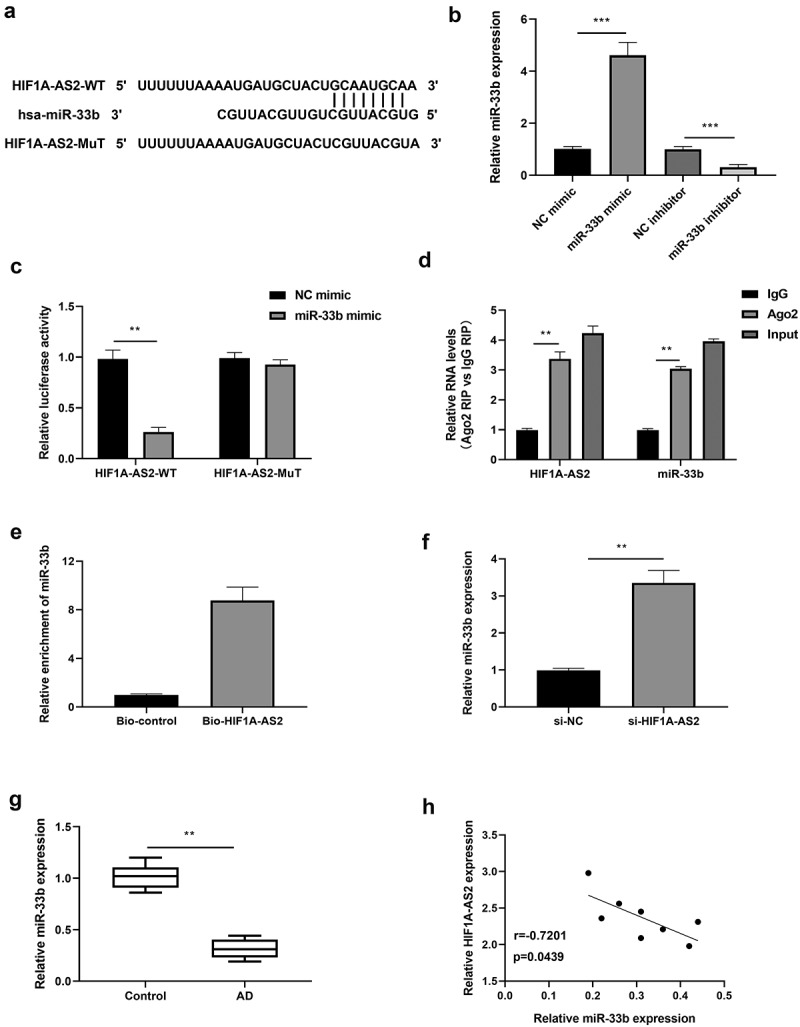
HIF1A-AS2 directly interacted with miR-33b in SMCs. (a) The binding sites of HIF1A-AS2-WT and HIF1A-AS2-MuT in the miR-33b were shown using starBase. (b) The expression of miR-33b was detected by RT-qPCR in SMCs treatment with miR-33b mimic or inhibitor. (c) The luciferase activity was measured in SMCs treatment with miR-33b mimic. (d) RIP analysis of HIF1A-AS2 or miR-33b expression in Ago2 and IgG group. (e) The targeting relations of HIF1A-AS2 and miR-33b were confirmed by RNA pull-down assay. (f) miR-33b expression in si-HIF1A-AS2-treated SMCs was measured by qRT-PCR. (g) miR-33b expression in AD tissues was detected by qRT-PCR. (h) Spearman correlation analysis of the correlation between HIF1A-AS2 and miR-33b. **P < 0.01, ***P < 0.001.

Previous studies have reported that miR-33b was negatively regulated by HIF1A-AS2 via targeting the binding seeds in osteosarcoma cells. Here, we investigated the relationship between HIF1A-AS2 and miR-33b in SMCs. As predicted by bioinformatics analysis, miR-33b was the miRNA that competitively bound with HIF1A-AS2 (their conserved binding sites shown in Figure 3(a)). Then, luciferase reporter, RIP, and RNA pull-down assays were implemented to verify their correlation. MiR-33b expression was over-expressed or knockdown by treatment with miR-33b mimic or inhibitor in SMCs, which was confirmed by qRT-PCR (Figure 3(b)). Results of Figure 3(c) showed that miR-33b mimic decreased the luciferase activity of the HIF1A-AS2 reporter vector, while there was no alteration in the mutated type of the vector. Results from RIP assay showed that HIF1A-AS2 and miR-33b were significantly enriched by the anti-Ago2 protein (Figure 3(d)). RNA pull-down assay further revealed that miR-33b bound biotin-labeled HIF1A-AS2 (Figure 3(e)). Accordingly, qRT-PCR assay displayed that miR-33b was increased markedly after treatment with si-HIF1A-AS2 (Figure 3(f)). Furthermore, miR-33b expression in human AD tissues was determined by qRT-PCR and results discovered a decreased expression of miR-33b in AD tissues (Figure 3(g)). In addition, there was a negative correlation between HIF1A-AS2 and miR-33b in AD tissues (Figure 3(h)). These results indicated that miR-33b directly targets the predicted 3'UTR of HIF1A-AS2.

### HIF1A-AS2 positively regulated HMGA2 expression by targeting miR-33b in SMCs

**Figure 4.**
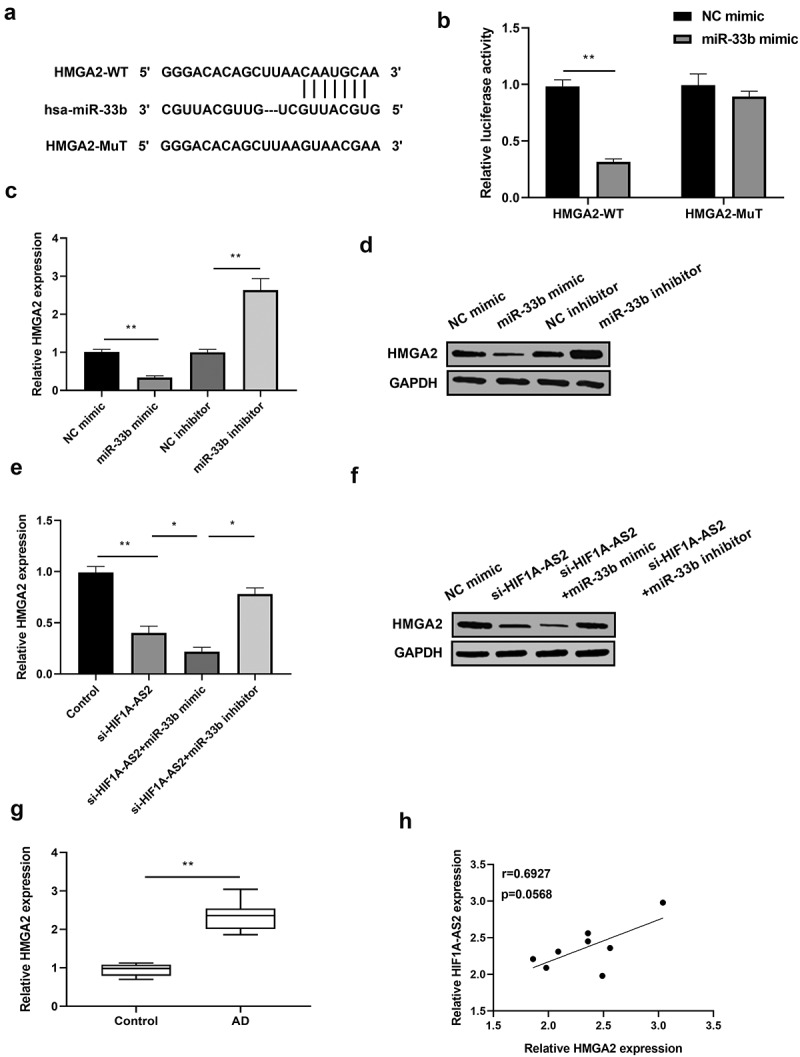
HMGA2 was a direct target of miR-33b and positively regulated by HIF1A-AS2. (a) The HMGA2 3'UTR-WT and HMGA2 3'UTR-MuT in the miR-33b binding sites were shown using TargetScan. (b) Luciferase activity of HMGA2 was measured in SMCs treatment with miR-33b mimic. (c) HMGA2 mRNA and (d) protein expression was detected in SMCs treatment with miR-33b mimic or inhibitor. (e) HMGA2 mRNA and (f) protein expression was measured in SMCs after transfection with si-HIF1A-AS2 or combined with miR-33b mimic or inhibitor. (g) HMGA2 expression was detected by qRT-PCR in AD tissues. (h) Spearman correlation analysis of the relationship between HIF1A-AS2 and HMGA2 in AD tissues. *P < 0.05, **P < 0.01.

As TargetScan predicted, HMGA2 might be the potential target of miR-33b. A site for miR-33b binding to the 3'UTR of HMGA2 was shown in Figure 4(a). Results from luciferase assay revealed that the luciferase activity of HMGA2-WT was inhibited in miR-33b mimic-transfected SMCs, but not in HMGA2-MuT (Figure 4(b)). In addition, HMGA2 expression was significantly decreased by miR-33b over-expression and increased by miR-33b knockdown, which was confirmed by Western blotting and qRT-PCR results (Figure 4(c,d)). Afterward, the relationship between HIF1A-AS2, miR-33b, and HMGA2 was analyzed. Results showed that HMGA2 expression was reduced by HIF1A-AS2 knockdown, and the reduction was further decreased by the incorporation of miR-33b mimic. However, the down-regulation of HMGA2 induced by HIF1A-AS2 knockdown was rescued by miR-33b inhibitor Figure 4(e,f). Subsequently, HMGA2 expression in human AD tissues was detected by qRT-PCR. As shown in Figure 4(g), HMGA2 was gradually elevated in AD tissues, relative to normal tissues. Of note, the relationship between HIF1A-AS2 and HMGA2 was positively in AD tissues (Figure 4(h)). These results demonstrated that HIF1A-AS2 positively regulated HMGA2 expression by targeting miR-33b.

### HIF1A-AS2 modulated SMCs proliferation, migration, and phenotypic switching via miR-33b/HMGA2 axis

**Figure 5.**
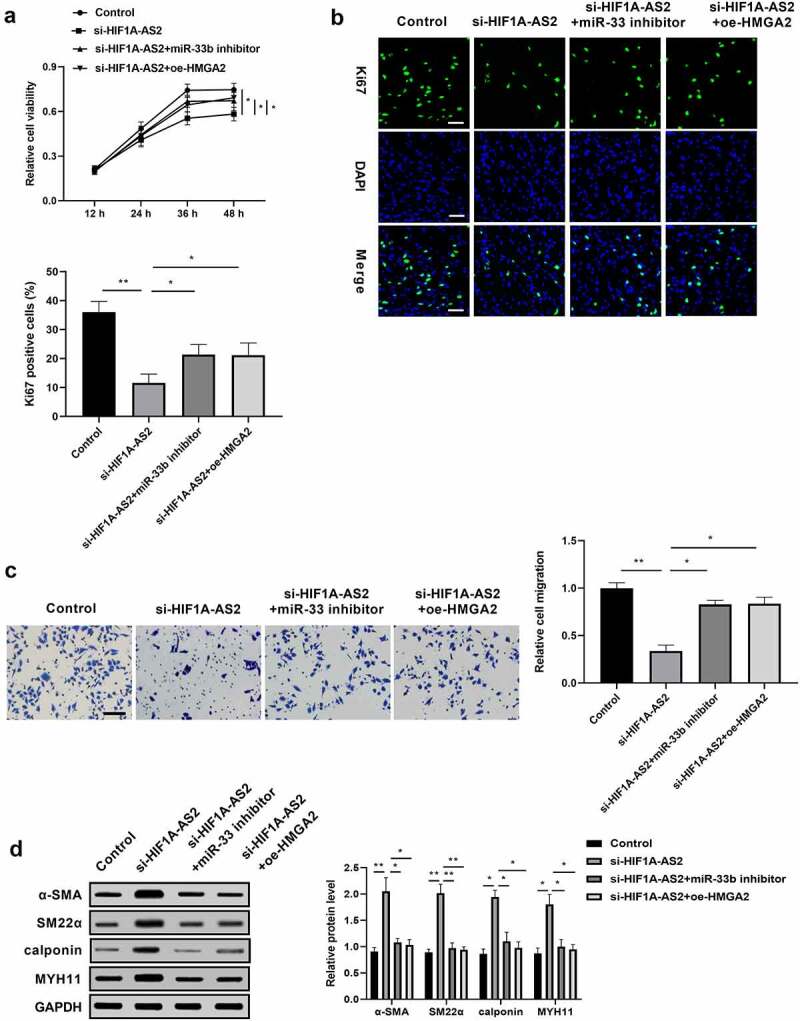
HIF1A-AS2 modulated SMCs proliferation, migration, and phenotypic switching via miR-33b/HMGA2 axis. SMCs were treated with si-HIF1A-AS2, meanwhile with miR-33b inhibitor or oe-HMGA2. (a) SMCs viability was detected by CCK-8 assay. (b) Ki67 positive cells were measured by Immunofluorescence. (c) The cell migration of SMCs was detected by transwell assay. (d) The protein levels of α-SMA, SM22α, calponin, and MYH11 were measured by Western blotting. *P < 0.05, **P < 0.01.

We have found that HIF1A-AS2 knockdown suppressed the proliferation and migration, and promoted phenotypic switching in SMCs, and identified the HIF1A-AS2/miR-33b/HMGA2 ceRNA mechanism by luciferase reporter analysis, RIP assay, and RNA pull-down assay. Next, a rescue experiment was performed to explore whether the inhibition effect of si-HIF1A-AS2 was mediated by miR-33b/HMGA2 axis. SMCs were transfected with si-HIF1A-AS2, or along with miR-362 inhibitor or oe-HMGA2 prior to PDGF-BB treatment. As Figure 5(a) shown, the viability of SMCs was decreased by HIF1A-AS2 knockdown, while the inhibition effect of HIF1A-AS2 knockdown was rescued by the incorporation of miR-33b inhibitor or oe-HMGA2. Moreover, silence of HIF1A-AS2 significantly reduced the fraction of Ki-67 SMCs, which was increased by down-regulation of miR-33b or up-regulation of HMGA2 (Figure 5(b)). Transwell migration assay discovered that the migration of SMCs was attenuated as HIF1A-AS2 was down-regulated, and the decrease was elevated by miR-33b mimic or oe-HMGA2 (Figure 5(c)). In addition, the expressions of α-SMA, SM22α, calponin, and MYH11 increased by si-HIF1A-AS2 in SMCs were repressed by inhibiting miR-33b or restoration of HMGA2 (Figure 5(d)). The rescue experiment proved that miR-33b/HMGA2 axis is essential for HIF1A-AS2 in regulation of SMCs proliferation, migration, and phenotypic switch.

## Discussion

AD is a cardiovascular emergency that seriously endangers human life. Although the majority of medical workers have made great efforts in the diagnosis and treatment of the disease, the incidence of the disease is increasing year by year. Therefore, it is urgent to explore the mechanism of AD and develop new treatment methods to prevent and treat AD. In this present study, HIF1A-AS2 was identified as a critical regulator in SMCs proliferation, migration, and phenotypic switch. HIF1A-AS2 was obviously increased in AD patients. Functional assay demonstrated that HIF1A-AS2 knockdown suppressed SMCs proliferation and migration, and promoted phenotypic switch though miR-33b/HMGA2 axis.

Generous studies discovered that HIF1A-AS2 is essential in modulation of cell proliferation, differentiation, and migration. Wu D et al. showed that inhibition of HIF1A-AS2 impeded trophoblast cell proliferation, migration, and invasion *in vitro* [17]. Another study showed that HIF1A-AS2 promotes human cardiomyocytes viability, migration, and invasion, which occurs in myocardial infarction [33]. Moreover, a study showed that HIF1A-AS2 is increased greatly in human umbilical vein endothelial cells in hypoxia and facilitates cell viability, migration ability, and tube formation [34]. However, the significance of HIF1A-AS2 in SMCs function and AD development has not been explored. Herein, this is the first report demonstrating that HIF1A-AS2 inhibition significantly decreases SMCs proliferation, migration, while increases phenotypic switch in mimic models of AD conditions *in vitro*.

LncRNAs, as ‘molecular sponges’ of miRNAs, regulate the expression of target genes by competitively binding mRNAs with miRNAs, which is one of the important mechanisms for their biological regulation [35]. The present study has identified that reduction of HIF1A-AS2 results in up-regulation of miR-33b. This data indicated a negatively correlation between miR-33b and HIF1A-AS2. This is probably associated with that miR-33b served as the target of HIF1A-AS2, as demonstrated by luciferase reporter, RIP, and RNA pull-down assays. MiR-33b was reported to be involved in cell proliferation and differentiation in human umbilical cord-derived stem cells [36]. It was also reported that miR-33b plays a critical role in the develpoment of atherosclerosis [37]. Rayner KJ et al. determined miR-33b as a promising therapeutic strategy for the treatment of dyslipidaemias that increase cardiovascular disease risk [38]. Based on these studies, we discovered that miR-33b exhibited a critical role in the development of AD modulated by HIF1A-AS2. Previous studies have reported that miR-33b modulates the proliferation, migration, and invasion of multiple tumors by inhibiting the expression of HMGA2 [^39–41^]. As a small nonhistone chromosomal protein, HMGA2 is normally highly expressed during embryogenesis whereas undetectable or lowly expressed in normal adult tissues, indicating that HMGA2 showed a critical role in cell proliferation and/or differentiation during embryonic development [42,43]. HMGA2 has been widely investigated in aortic dissection, and has been found to regulate rat aortic vascular SMCs viability and apoptosis [44,45]. Consistent with the above studies, we discovered that HMGA2, as a target of miR-33b, is involved in SMCs proliferation, migration, and phenotypic switch.

There were limitations in our study. First, the Spearman’s correlation experiments was not robust due to the relatively small sample size. Second, the animal model of AD was not performed, which can better clarify the functional role and mechanisms of HIF1A-AS2 in the development of AD. Third, the targets of HIF1A-AS2 are diverse, it is necessary to further study the molecular mechanisms of AD using *in vitro and in vivo* studies, which will provide a potential novel strategy for AD.

## Conclusions

In summary, our results clarify for the first time that HIF1A-AS2, as a ceRNA for miR-33b, inhibits SMCs proliferation and migration, and facilitates phenotypic switch through regulating HMGA2, which provides a new insight into the molecular mechanism of HIF1A-AS2 in the development of AD, and shed light on a potential target for the treatment of AD.
